# Patients Understanding of their Diagnosis and Treatment Plans During Discharge in Emergency Ward in a Tertiary Care Centre: A Qualitative Study

**DOI:** 10.31729/jnma.4639

**Published:** 2019-10-31

**Authors:** Ajay Kumar Yadav, Shyam Sundar Budhathoki, Masum Paudel, Ritesh Chaudhary, Vijay Kumar Shrivastav, Gyanendra Bahadur Malla

**Affiliations:** 1 Department of General Practice and Emergency Medicine, B P Koirala Institute of Health Sciences, Dharan, Nepal; 2 Golden Community, Lalitpur, Nepal

**Keywords:** *emergency*, *health literacy*

## Abstract

**Introduction:**

Many patients do not understand their emergency care plan or their discharge instructions. Patients should understand both the care that they received and their discharge instructions. Patients’ knowledge of the diagnosis and treatment plan is an integral component of patient education. The objective of the study is to identify and describe the areas of patients’ understanding and confusion about emergency care and discharge instructions at the BP Koirala Institute of Health Sciences, Dharan, Nepal.

**Methods:**

A qualitative study involving 426 patients discharged from the emergency unit of BPKIHS using a semi-structured questionnaire. Cases who are Leaving against medical advice, absconded cases and those patients who came just for vaccination are excluded from the study. The ethical approval for this study was received from the institutional review committee of BP Koirala Institute of Health Sciences, Dharan [Ref: IRC/0752/016].

**Results:**

There were 256 (60.1%) of men in this study. More than half of the participants reported not being able to read English. More than 90% of the respondents reported they could not read their prescription at all. While patient could point out their understanding of their diagnosis at discharge, most of them could not tell the names and the dosage of all the drugs prescribed to them at discharge. More than 95% of the patients could not tell the most common side effects of the drugs that they are prescribed.

**Conclusions:**

There is a need to further explore the factors influencing the understanding of the patients regarding their treatment plan. Interventions to understand the health literacy needs and ways to improve the health literacy of the patients are needed.

## INTRODUCTION

Communication among the physician, nurse and health care assistant as a team, and patient is essential for effective delivery of healthcare.^1^ Patients do not understand their disease conditions, plan of care at the hospital and after discharge.^2^ This leads to an increase in adverse events following discharge.^3^ Patients’ understanding of their plan of care affects their ability to assume self-care after discharge.^4^ Health professionals overestimate patients’ understanding of treatment plans and diagnosis during discharge.^5^

Understanding backgrounds and health literacy needs are critical to ensure patient’s understanding of their health conditions and planned care for better patient outcomes.^6-8^ Poor comprehension of instructions and inadequacies of communication between the patient and physician contribute to patient noncompliance.^9,10^ Research on the understanding and confidence of patients their comprehension of diagnosis is essential.^11^

With limited literature found, we chose to conduct a study to explore patients understanding of their diagnosis and treatment plans at discharge.

## METHODS

This is a qualitative study conducted among all discharge cases from the emergency ward of BPKIHS. The study participants were taken using consecutive sampling method between Nov 2016-January 2017. The study was conducted after approval from the institutional review committee (IRC) of B P Koirala Institute of Health Sciences, Dharan [Ref:IRC/0752/016]. Informed consent was taken. Data regarding personal identification were not taken.

We took 426 discharged patients from the emergency ward of BPKIHS for participation in the study. A semi-structured questionnaire was used to collect the data regarding socio-demographic variable, knowledge & understanding of treatment & discharge summary. Qualified General Practitioners of BPKIHS conducted the research. The questionnaire was prepared based on variable literatures and the opinion/input of senior academicians of general practice at BPKIHS. The questionnaire was pretested at the general outpatient clinic of BPKIHS. All discharge cases at emergency ward of BPKIHS willing to participate took part in a 15-25 minutes interview. Cases who are Leaving against medical advice, absconded cases and those patients who came just for vaccination were excluded.

The response was recorded in excel sheet and analysed in SPSS. Any data linking to patient identity were removed and coded to maintain patient’s privacy. Descriptive statistics were used for analysis and expressed in frequency and percentages.

Interview was done using a semi-structured questionnaire was to collect the data regarding Socio-demographic variable, knowledge & understanding of treatment & discharge summary. There were no missing data. The potential of recall bias was minimized by having the interview immediately during the discharge and the potential of respondents bias was minimized by having the interview taken by a non-treating member of the team without wearing the white coat.

## RESULTS

Among the 426 patients included in this study, most patients were between the age of 25-59 years of age. There were 256 (60.1%) men in this study. More than half of the participants reported not being able to read English. A total of 397 (93.2%) of the respondents reported they could not read their prescription at all (Table 1).

**Table 1:** Socio demographic characteristics of the patients.

Characteristics		n (%)
Sex	Male	256 (60.1)
	Female	170 (39.9)
Age (years)	15-24	61(14.3)
	25-59	285 (66.9)
	60 and above	80 (18.8)
	Did not go to school	52 (12.2)
	Primary	78 (18.3)
Education	Secondary	133 (31.3)
	Higher Secondary	117 (27.5)
	University	46 (10.7)
Ability to read English	Yes	131 (30.8)
	No	295 (69.2)
Ability to read prescription	Yes	29 (6.8)
	No	397 (93.2)

The diagnosis of the patients when categorized into system wise conditions, the highest numbers are 92 (21.6%) gastrointestinal and 82 (19.2%) respiratory conditions (Table 2).

**Table 2:** Primary diagnosis of the patients.

Diagnosis	n (%)
Respiratory Conditions	82 (19.2)
Cardiovascular conditions	51 (12.0)
Injuries/RTA	66 (15.5)
Neurological conditions	36 (8.5)
Gastrointestinal conditions	92 (21.6)
Urogenital conditions	40 (9.4)
Others	59 (13.8)

While patient could point out their understanding of their diagnosis at discharge, most of them could not tell the names and the dosage of all the drugs prescribed to them at discharge. There were 409 (97.1%) of the patients who could not tell the most common side effects of the drugs that they are prescribed (Table 3).

**Table 3:** Understanding of the discharge summary by the patients.

Characteristics		n (%)
Frequency of Emergency Visit) In past 1 year(	Once	299 (70.2)
	Twice	72 (16.9)
	Thrice	39 (9.2)
	Four Times	16 (3.7)
Understood the disease condition	Yes	246 (57.7)
	No	180 (42.3)
Can name the drugs prescribed	Yes	32 (7.3)
	No	394 (92.7)
Frequency and duration days (of the drug	Yes	225 (52.8)
	No	101 (47.2)
Routes of drug administration	Yes	283 (66.4)
	No	143 (33.6)
Understand the common side effects of the drugs	Yes	17 (3.9)
	No	409 (97.1)
Understand the dietary modification with treatment	Yes	136 (31.9)
	No	290 (68.1)
Know the follow-up plan	Yes	339 (79.5)
	No	87 (20.5)

## DISCUSSION

Understanding whether the patients are able to comprehend their diagnosis and treatment plan is essential for better service and the patient’s outcome. emergency wards in hospitals have excessive patient turnover and patients discharged are generally offered a routine follow-up in out-patients for further clinical care. There were 52% of discharged emergency department patients having education less than secondary level, while all the written material for patients was written in English with the use of technical terms. While 10% of patients reported having a university education, only 6% of the patients reported they could read the prescriptions and only 30% could read English sentences. Discrepancy between the reading level of written instructions and patients reading ability may be a significant contributor to patient noncompliance.^8^

Many patients do not understand their emergency care plan or their discharge instructions. Patients should understand both the care that they received and their discharge instructions.^12^ While about half of the patients expressed that they understood their disease conditions, only 7% could name their drugs and 3% said they knew about the side effects of the drugs prescribed. This clearly indicates the need of interventions to understand how this gap of physician patient communication can be filled. The quality of discharge planning is an important determinant of patient outcomes following hospital discharge. Patients often report inadequate discussion prior to discharge regarding major elements of the post discharge treatment plan, including medication and daily activities. Patients knowledge of the diagnosis and treatment plan is an integral component of patient education and is a central part of the patient’s rights. Interventions like those that printed discharge notes may help patients who can read English to understand their discharge summary. The findings from this study is relevant to tertiary hospitals and teaching hospitals all over Nepal.

The study has many limitations. Firstly, being a descriptive study, it is not able to highlight factors responsible for the lack of understanding of the diagnosis and treatment plans among the patients. Another limitation is that the study was conducted in an acute care setting of the hospital which has limited time for interaction between patients and doctors and thereby researchers, which limits further exploration of the research questions. However this study is able to highlight that there is a huge proportion of patients that do not understand their diagnosis and the treatment plan and recommend further research to address this issue.

## CONCLUSIONS

There is limited ability to read prescriptions and even the negligible ability to understand the diagnosis among the patients at the emergency ward. There is a need to further explore the factors influencing the understanding of the patients regarding their treatment plan. Interventions to increase the health literacy needs and ways to improve the health literacy of the patients are needed.
